# Multiorgan Toxicity from Dual Checkpoint Inhibitor Therapy, Resulting in a Complete Response—A Case Report

**DOI:** 10.3390/medicina60071129

**Published:** 2024-07-12

**Authors:** Skaistė Astašauskaitė, Rita Kupčinskaitė-Noreikienė, Inga Zaborienė, Rūta Vaičiūnienė, Tomas Vanagas, Darius Pranys, Lina Poškienė, Elona Juozaitytė

**Affiliations:** 1 Institute of Oncology, Medical Academy, Faculty of Medicine, Lithuanian University of Health Sciences, LT-50161 Kaunas, Lithuania; 2 Department of Radiology, Medical Academy, Faculty of Medicine, Lithuanian University of Health Sciences, LT-50161 Kaunas, Lithuania; 3 Department of Nephrology, Medical Academy, Faculty of Medicine, Lithuanian University of Health Sciences, LT-50161 Kaunas, Lithuania; 4 Department of Surgery, Medical Academy, Faculty of Medicine, Lithuanian University of Health Sciences, LT-50161 Kaunas, Lithuania; 5 Department of Pathology, Medical Academy, Faculty of Medicine, Lithuanian University of Health Sciences, LT-50161 Kaunas, Lithuania

**Keywords:** immune checkpoint inhibitors, immune checkpoint inhibitor-induced colitis, immune-mediated nephritis, immune checkpoint inhibitor-induced hepatitis, clear cell renal carcinoma, complete response

## Abstract

Immunotherapy treatment with checkpoint inhibitors (ICIs) has led to a breakthrough in the treatment of oncological diseases. Despite its clinical effectiveness, this treatment differs from others, such as cytotoxic chemotherapy, in that it causes immune-related adverse events. This type of toxicity can affect any organ or organ system of the body. We present a literature review and a rare clinical case from our clinical practice, in which a patient with metastatic clear cell renal carcinoma was treated with a single dose of dual checkpoint blockade (cytotoxic T-lymphocyte-4 (CTLA-4) and programmed death-1 (PD-1)) and simultaneously diagnosed with colitis, hepatitis, and nephritis. After early immunosuppressive treatment with the glucocorticoids, complete organ function recovery was achieved. The follow-up revealed a sustained complete response lasting more than a year.

## 1. Introduction

Immunotherapy treatment with checkpoint inhibitors (ICIs) has led to a breakthrough in the treatment of oncological diseases. Based on response in several tumor types, immunotherapy has produced durable responses in selected patients, and in rare cases even complete response can be achieved in metastatic disease [1,2]. The mechanism of action is to stimulate the immune system against tumor cells by blocking specific receptors, including cytotoxic T-lymphocyte-4 (CTLA-4) and programmed death-1 (PD-1), or its ligand (PD-L1) [3]. Renal carcinoma is one of the solid tumors that is sensitive to immunotherapy. Despite its clinical effectiveness, there are many side effects that differ from other treatments, such as cytotoxic chemotherapy, which may affect any organ or system of the body [4]. Toxicity is graded on the Common Terminology Criteria for Adverse Events (CTCAE), and grades ≥3 are defined as severe adverse events (AEs). Immune-related AEs (irAEs) are common and have been reported to occur in 90% of patients treated with anti-CTLA-4 and 70% of patients treated with anti-PD-1/PD-L1. In a systematic review of 50 trials, the incidence of grade 3/4 irAEs was 21% [3]. Most common irAEs are dermatological (itching or rash), followed by gastrointestinal (diarrhea, colitis, hepatitis), endocrinopathies (hypo- and hyperthyroidism, hypophysitis; less common—adrenal insufficiency, diabetes), and musculoskeletal. Uncommon irAEs are pneumonitis, myocarditis, neurotoxicity, nephritis, and hematological toxicity—they have a higher potential to be severe and even fatal, with mortality rates ranging between 10% and 17%. The mortality rate for myocarditis is extremely high at 39.7% [5]. The strategy of using combined ICI blockage is associated with a higher frequency, wider spectrum, and earlier timing of irAEs [6]. The good news is that several publications have demonstrated a positive association between the development of irAEs and anti-tumor responses to ICI across some tumor types [2]. This is also evident in our patient’s case.

## 2. Case Report

**Figure 1:**
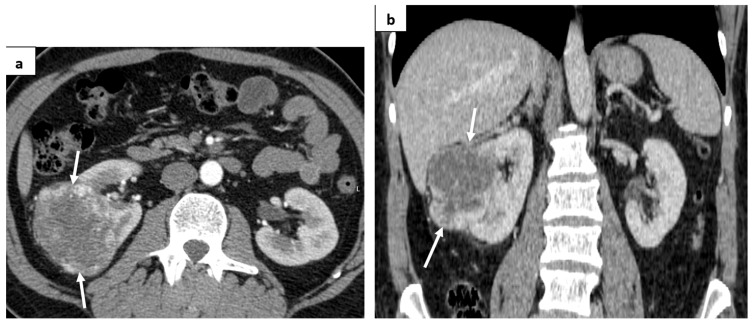
CT images: (**a**) axial plane; arterial phase—a hypervascular tumor in the right kidney was detected (white arrows); (**b**) coronal plane.

A 48-year-old male patient was admitted to our hospital due to a right renal tumor, which was found during an ultrasound (US) examination. Computed tomography (CT) was performed, showing a 7.8 × 7.7 cm hypervascular tumor with a central part of necrosis on the right kidney. No distant metastases were detected (Figure 1).

**Figure 2:**
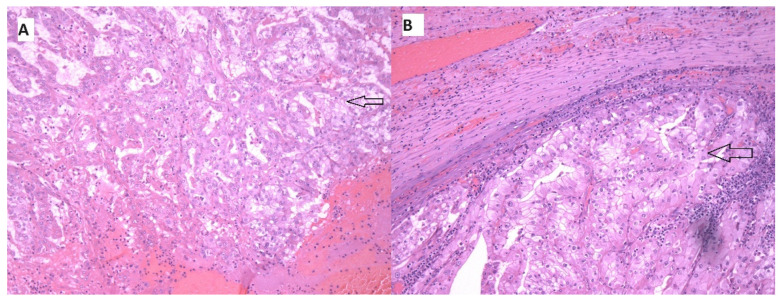
(**A**) Clear cell RCC primary tumor (black arrow) immunohistochemical stain HE; (**B**) metastasis of clear cell RCC (black arrow) immunohistochemical staining with HE.

**Figure 3:**
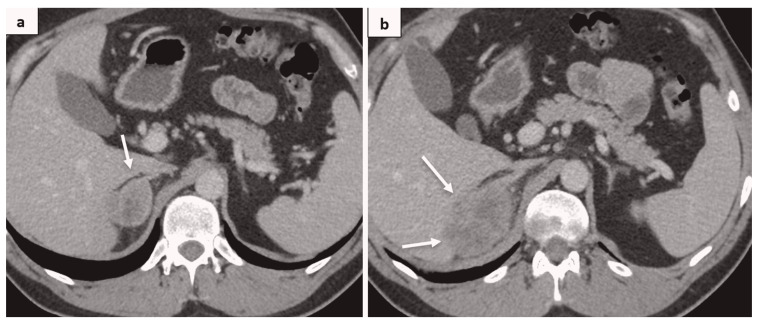
(**a**) A solid mass in the right adrenal gland is depicted (white arrow); (**b**) local recurrence, infiltrating S6 of the liver (white arrows).

The patient underwent laparoscopic right nephrectomy in November 2020. The postoperative pathological diagnosis was clear cell renal carcinoma pT2aR0G2 (Figure 2A). The patient felt well, and surveillance was suggested according to protocol. Every 6 months, CT was performed, and no signs of tumor relapse were detected. In January 2022, CT showed a local recurrence of the tumor, which was 7.1 × 5.3 cm in size, with close contact to the right crus of diaphragm, growing into the liver parenchyma in S6, as well as infiltrating the right adrenal gland (Figure 3).

**Figure 4:**
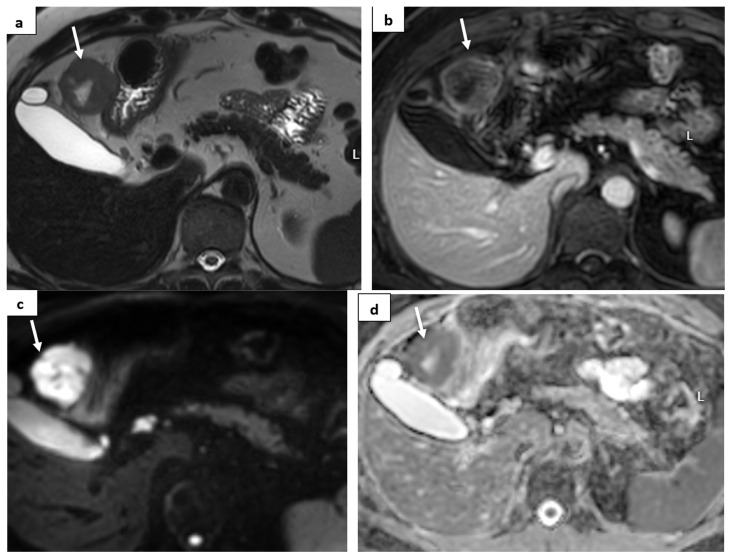
**MRI images**: (**a**) T2W image—a solid lesion with a central part of necrosis is shown (arrows); the lesion is hypervascular after c/m administration (**b**); diffusion-weighted image (DWI) with a high b value (**c**)—the tumor shows restricted diffusion and a low apparent diffusion coefficient (ADC) value on ADC map (**d**).

In March 2022, the patient underwent a radical extirpation of the recurrent tumor and right adrenalectomy. Pathological findings coincided with primary diagnosis—clear cell renal carcinoma (Figure 2B). In February 2023, a CT showed a subhepatic recurrence of the tumor under the left liver lobe; a solid mass ~3.6 cm in size was detected. According to magnetic resonance imaging (MRI), a 3.7 × 4.1 × 4 cm subhepatic tumor was depicted near the pyloric part of the stomach and the lateral surface of the duodenum; another tumor 0.8 cm in size was depicted in the mesentery. The images are shown in Figure 4.

The multidisciplinary team (MDT) discussed the case, assessed it, and classified it as an intermediate risk in line with the Heng criteria. Therefore, immunotherapy treatment was recommended. The patient had no symptoms of disease and before the systemic treatment his condition was rated as good, ~80% according to the Karnofsky performance status scale. The blood tests showed normal liver and kidney function (creatinine—115 µmol/L, total bilirubin—6.4 µmol/L, direct bilirubin—1.97 µmol/L, serum alanine aminotransferase (ALT) 49 U/L, aspartate aminotransferase (AST) 36 U/L, C-reactive protein 34 mg/L). On 28 March 2023, a first dose of combined immunotherapy was administered: nivolumab at a dose of 3 mg/kg and ipilimumab at a dose of 1 mg/kg i/v. Ten days after the first immunotherapy dose, the patient developed fever and oliguria. After 3 days of self-treating, he was admitted to the regional hospital. The patient developed new symptoms—abdominal pain, diarrhea, jaundice, anuria. Blood tests showed an acute renal failure (creatinine—777 µmol/L) and liver injury (total bilirubin—97 µmol/L, direct bilirubin—63.5 µmol/L, ALT 87 U/L, AST 74 U/L, high C-reactive protein (334 mg/L). Hepatitis viral markers were negative. Liver US revealed homogeneous liver architecture, and no focal lesions or evidence of obstruction. Blood and urine culture cl. difficile toxins tests were negative, antibacterial treatment with meropenem was started, and an urgent hemodialysis performed. Subsequently, the patient was transported to our hospital’s nephrology department. After multidisciplinary consultations, it was concluded that colitis (grade 2), hepatitis (grade 3), and acute renal injury (AKI, grade 4) were immunotherapy related, and acute renal insufficiency was caused by acute interstitial nephritis. Treatment with steroids was initiated. The patient received methylprednisolone 1 mg/kg/day 1 week, with dose reduction every 3 days after that. Hemodialysis was performed twice more. A liver biopsy was considered but not performed as a rapid improvement in their liver function was observed after the initiation of steroids. Once the steroids treatment started, the patient’s condition dramatically improved and he was discharged from the hospital 19 days after admission with oral methylprednisolone, which was tapered off over 6 weeks.

**Figure 5:**
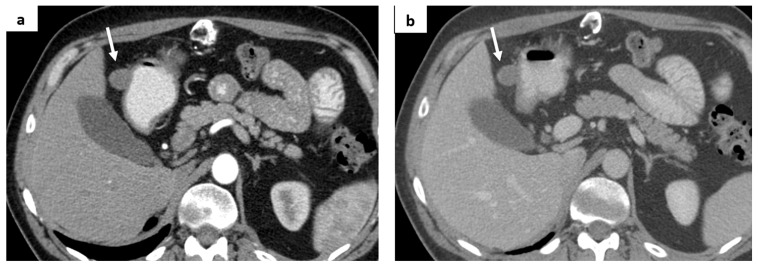
CT images, axial plane; arterial (**a**) and portal venous phase (**b**); a subhepatic residual solid mass is depicted (white arrows).

**Figure 6:**
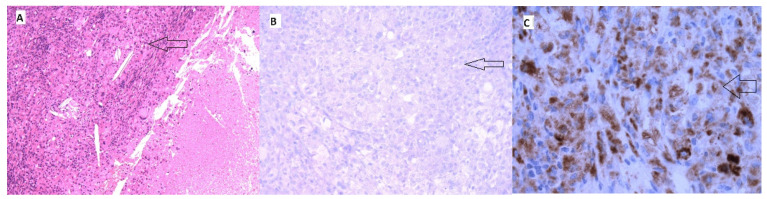
Necrosis without evidence of carcinoma cells after a single dose of double immunotherapy (black arrows). (**A**) Immunohistochemical staining with HE; (**B**) immunohistochemical staining with PAX8; (**C**) immunohistochemical staining with CD68.

In August 2023, CT showed a residual mass in the subhepatic space, ~1.7 × 1.5 cm (Figure 5). In September 2023, laparoscopic extirpation of the residual tumor was performed. Pathological assessment found necrosis only (Figure 6). Systemic treatment was not performed again. A follow-up CT revealed a continuous complete response to one dose of combined immunotherapy, lasting now for over a year.

## 3. Discussion

**Immune-mediated colitis and diarrhea:** Gastrointestinal irAEs can occur anywhere in the gastrointestinal tract, including the esophagus, stomach, small intestine, colon, gallbladder, bile ducts, and liver [7,8]. ICI-associated diarrhea and colitis are defined by the American Society of Clinical Oncology (ASCO) based on symptoms alone and not on colonic inflammation. Diarrhea describes watery frequent stools which can be accompanied by colitis symptoms (abdominal pain, fever, and mucus or blood in the stools) [9]. Although CTCAE ICI-related diarrhea and colitis are classified separately, these conditions (and their management) overlap. Patients who receive ICI and experience diarrhea should be considered very seriously because undiagnosed and untreated, this condition can lead to a life-threatening complication—perforation, even though, according to the literature, the risk of ICI-induced perforation is about 0.6% [10]. The incidence of colitis is 3.4–22% with CTLA-4 inhibitor therapy, 0.7–12.8% with combined CTLA-4 and PD-1 inhibitor therapy, and 0.7–2.6% with PD-1/PD-L1 inhibitor monotherapy [11,12]. It is known that the risk and severity of the ICI-induced colitis can depend on the primary tumor (more common for melanoma) [13], composition of the intestinal microbiome [14], lack of vitamin D [15], and the use of nonsteroidal anti-inflammatory drugs (NSAIDs) [16]. The timing of the event of clinical manifestation is different but, in most cases, it develops from a few weeks to a few months after ICI initiation and sometimes it can occur even years after the completion of immunotherapy [17]. The clinical and radiological pictures of ICI-induced colitis and inflammatory bowel disease can be similar. CT scans show findings such as mesenteric vessel engorgement, bowel wall thickening, and fluid-filled colonic distention [18]. Other causes can also be associated with diarrhea and colitis. In order to exclude them, patients should be tested for hyperthyreosis and various infectious agents (C. difficile, parasites, CMV, etc.). If diarrhea symptoms are grade 2 or higher, or accompanied by apparent colitis symptoms, holding ICI until recovery to grade 1 and corticosteroids at 1–2 mg/kg/day is the first-line option for treatment [4].

**Immune-mediated hepatotoxicity:** Immunotherapy-related acute hepatitis is rare, but it can lead to acute liver failure. Hepatitis occurs in 5–10% of patients during treatment with ICI as a monotherapy (~2% grade 3) and 25–30% during treatment with a combination of anti-PD(L)-1 and anti-CTLA-4 (~15% grade 3) [19]. Hepatitis usually occurs 6–14 weeks after the initiation of treatment [4]. A recent study showed a significantly earlier onset of hepatitis in patients treated with anti-CTLA-4 antibodies compared to those receiving anti-PD-1/PD-L1 antibodies [20]. The exact mechanisms of irAEs are under investigation and are not fully understood. T-cell activation occurs, CD4+ helper T-cells secrete many cytokines, and CD8+ T-cells infiltrate the tissue. A liver biopsy tissue often reveals immune-mediated liver injury with focal or confluent necrosis and a marked infiltration of activated T-cell lymphocytes [21]. Hepatitis often is asymptomatic; therefore, clinical guidelines recommend checking AST, ALT, and bilirubin levels before each cycle. If any deviations from the normal ranges are detected, a differential diagnosis should be conducted to exclude other possible causes of liver damage (metastases, viral hepatitis, drugs, alcohol consumption, etc.). Liver damage with elevated bilirubin usually indicates severe tissue damage, which carries a high risk of liver failure and requires more aggressive treatment [22]. Patient education plays an important role in recognizing symptoms like jaundice, dark urine, pruritus, fatigue, and abdominal discomfort, although the presence of these symptoms corresponds with more severe cases [23]. Imaging findings are nonspecific, and CT can show hepatomegaly, periportal edema, and periportal lymphadenopathy, depending on the severity of the liver injury [24]. It is recommended that grade 2 or higher hepatotoxicity is treated with corticosteroids ((1–2 mg/kg/day (methyl)prednisolone) according to severity), tapering the dose within 6–8 weeks until discontinuation [25]. If persistent or if treatment refractory hepatitis occurs, liver biopsy should be considered for the exclusion of other etiologies and alternative immunosuppressive therapy should be considered [4]. Restarting therapy with ICI after immune-related hepatitis is controversial. Most society guidelines recommend the continuation of therapy for grade 1 toxicity, rechallenging of therapy in grade 2 toxicity, and permanent discontinuation for grade 3 and 4 patients. However, some studies have shown tolerance of retreatment with ICI, even with grade 3 hepatotoxicity [24].

**Immune-related renal injury** is less common than skin, gastrointestinal, and endocrine toxicities. Combination therapy with CTLA-4 and PD-1 inhibitors was found to be associated with a higher incidence of immune-related nephritis (4%) compared to monotherapy (approximately 2%) [26]. The median time to immune-related nephrotoxicity is approximately 14–16 weeks after the first dose. AKI was diagnosed 2 weeks after the initiation of treatment in the case we have presented. Despite the low frequency of adverse events, it can lead to serious consequences: renal failure requiring organ replacement therapy; dialysis, which occurred in our patient; and this prolonged and undiagnosed condition can also lead to chronic renal failure. The precise mechanism of ICI-related nephritis is unknown, but there are a few theories on this basis. One of the mechanisms is the development and proliferation of auto-reactive T-cells. There are data on checkpoint receptor expression in non-tumor tissues, where low PD-L1 is expressed in renal tubular cells, and ICI can induce inflammatory damage through increased production renal and generation of pro-inflammatory cytokines [27,28]. In two multicenter retrospective studies, the outcomes in patients with ICI-related AKI are generally favorable, with 64% to 85% reporting renal disease recovery, but the percentage drops sharply to 46% when renal replacement therapy is required [29,30]. The treatment strategy for irAEs is the administration of corticosteroids. The timing of the initiation of corticosteroid therapy may also have a significant impact on the recovery of renal function. In the literature, the initiation of corticosteroid therapy within 14 days of diagnosis of ICI-related AKI was also associated with a higher likelihood of recovery (OR 2.64; 95% CI: 1.58–1.58). 4.41) [29,30].

We present our patient’s case to show the importance of the early recognition of severe irAEs. After starting treatment with combination ICI, awareness should be held because fatal irAEs often arise early after initiating treatment, at a median of 15 days, which is much earlier than most non-fatal events [31]. According to these data, some authors suggest close monitoring of the patient during the first dual ICI cycle (clinical and laboratory assessment every week for the first 3–4 weeks) [31]; still, these recommendations lack evidence. Most guidelines recommend monitoring before every treatment cycle (clinical assessment and blood tests to evaluate possible kidney, liver, and other vital organs function), patient education, and early treatment if irAEs are suspected. If the treatment of irAEs is needed, steroids are the medication of the first choice, with 1–2 mg/kg/day (methyl)prednisolone according to severity.

## 4. Conclusions

In conclusion, our case shows that severe immune-related adverse events can occur early, even after the first and only dose of ipilimumab and nivolumab. One cycle of dual-agent immunotherapy was sufficient to achieve a complete response. Considering new systemic options for the treatment of oncological diseases, we can radically improve the prognosis of our patients, but it is necessary to be aware of a completely different toxicity profile of the adverse events. Early recognition and timely, appropriate treatment allow for a better preservation of organ functions, reduce the need for surgical interventions, shorten hospitalization time, and improve the quality of life for patients.

## Data Availability

The data presented in this study are available upon request from the corresponding author.
